# Oleaginous yeasts- substrate preference and lipid productivity: a view on the performance of microbial lipid producers

**DOI:** 10.1186/s12934-021-01710-3

**Published:** 2021-12-07

**Authors:** Pariya Shaigani, Dania Awad, Veronika Redai, Monika Fuchs, Martina Haack, Norbert Mehlmer, Thomas Brueck

**Affiliations:** Werner Siemens-Chair of Synthetic Biotechnology (WSSB), Technical University of Munich, Lichtenbergstraße 4, 85748 Garching, Germany

**Keywords:** Oleaginous yeasts, Carbon substrate preference, Fermentation inhibitor tolerance, Biomass yield, Lipid yield, Complex lignocellulosic/marine biomass hydrolysate

## Abstract

**Background:**

Oleaginous yeasts are promising microbial platforms for sustainable, bio-based production of biofuels and oleochemical building blocks. Bio-based residues provide sustainable and cost-effective carbon sources for fermentative yeast oil production without land-use change. Considering the regional abundancy of different waste streams, we chose complex biomass residue streams of marine origin; macroalgae hydrolysate, and terrestrial origin; wheat straw hydrolysate in the presence, and absence of corn steep liquor as a complex nitrogen source. We investigated the biomass and lipid yields of an array of well-described oleaginous yeasts; *R. glutinis*, *T. asahii*, *R. mucilaginosa*, *R. toruloides, C. oleaginosus* growing on these hydrolysates. Furthermore, their sugar utilization, fatty acid profile, and inhibitory effect of the hydrolysates on yeast growth were compared. For correlative reference, we initially performed comparative growth experiments for the strains on individual monomeric sugars separately. Each of these monomeric sugars was a dominant carbon source in the complex biomass hydrolysates evaluated in this study. In addition, we evaluated N-acetylglucosamine, the monomeric building block of chitin, as a low-cost nitrogen and carbon source in yeast fermentation.

**Results:**

*C. oleaginosus* provided the highest biomass and lipid yields. In the wheat straw and brown algae hydrolysates, this yeast strain gained 7.5 g/L and 3.8 g/L lipids, respectively. Cultivation in algae hydrolysate resulted in a higher level of unsaturated fatty acids in the lipids accumulated by all yeast strains. *R. toruloides* and *C. oleaginosus* were able to effectively co-utilize mannitol, glucose, and xylose. Growth rates on wheat straw hydrolysate were enhanced in presence of corn steep liquor.

**Conclusions:**

Among the yeast strains investigated in this study, *C. oleaginosus* proved to be the most versatile strain in terms of substrate utilization, productivity, and tolerance in the complex media. Various fatty acid profiles obtained on each substrate encourage the manipulation of culture conditions to achieve the desired fatty acid composition for each application. This could be accomplished by combining the element of carbon source with other formerly studied factors such as temperature and oxygen. Moreover, corn steep liquor showed promise for enhancement of growth in the oleaginous strains provided that carbon substrate is available.

**Supplementary Information:**

The online version contains supplementary material available at 10.1186/s12934-021-01710-3.

## Background

Growing world population and climate change, combined with overused fossil resources are driving the development of sustainable bio-based processes. First generation bioprocesses propagated the use of edible plant oils and starch for the generation of biofuels, chemical building blocks, cosmetics, and pharmaceuticals. However, the increasing use of edible oils for non-food applications has led to significant land use change and an associated loss of biodiversity, exemplified by the ever-growing demand for palm oil [1–5].

Oleaginous yeasts are promising microbial platforms for sustainable, bio-based production of oleochemical building blocks and biofuels [6, 7]. To that end, oleaginous yeasts allow oil production with short production cycles independent of geographical, seasonal, and climate limitations [8, 9]. Furthermore, they can utilize low-value substrates and turn them into valuable triglycerides (TAGs) [10]. Yeast triglyceride oil product is chemically equivalent to plant oil resources, while its production does not induce land use change or compete with any agricultural activity [11].

Nevertheless, it is inefficient to implement the industrial use of yeast oils (YOs) without a sustainable and cost-effective yeast bioprocess. The cost of the fermentation medium and a high oil yield are two of the main challenges in the techno-economic feasibility of YO production [11, 12]. Global regions (for instance Ukraine, US, Argentina, and Russia) with access to large agricultural lands generate large quantities of terrestrial lignocellulosic residues, and on the coast, marine biomass could be considered as the renewable and cheap feedstocks. Advanced bioprocesses enable the use of bio-based residues without land use change to generate commodity products and fine chemicals. For oleochemicals, this pertains particularly to the use of complex biomass residues, such as cereal straw, wood waste, or algae as a fermentation feedstock for oleaginous yeasts [11]. Therefore, the mild enzymatic hydrolysis of forestry- and agro-industrial residues, to liberate fermentable sugars, has attracted a lot of attention in that regard [4]. These waste biomass hydrolysates have been flagged as sustainable and low-cost carbon sources for fermentative YO production. Using these hydrolysates would enhance the economic and ecological efficiency of the process and would eliminate further deforestation and biodiversity loss by expanding plant oil production [13].

A promising sustainable feedstock for biorefinery purposes is algae, highly available as marine biomass. The benefits of this type of raw material are fast growth, high availability of alluvial biomass, and no competition with agricultural land [14]. Compared to the terrestrial plant biomass, marine residues have a 6–10 times higher area productivity and lack lignin, which eliminates the need for pre-treatments and provides for simplified enzymatic hydrolysis, both aspects increasing the energy, economic and ecological efficacy of the entire bioprocess [10, 11].

In addition, an abundant and sustainable lignocellulosic agricultural residue of terrestrial origin is wheat straw, which has also been established as straw hydrolysates for the production of biofuels, such as bioethanol or yeast-derived biodiesel [15, 16]. The worldwide estimation of wheat residues production in the year 2012 was 887 million tons, from which 400 million tons of wheat straw remained unused after all other applications [17]. A further important agro-industrial feedstock is corn steep liquor, a by-product of the corn wet-milling industry. Since 1909 it has attracted attention as an inexpensive alternative source of organic nitrogen and vitamins to supplement the fermentation medium [18–20]. This feedstock contains a considerable amount of water-soluble vitamins, polypeptides, and amino acids, which are great sources of organic nitrogen, as well as minerals cumulatively acting as growth stimulants [21].

In this study, five wellknown oleaginous yeast strains *Rhodotorula glutinis*, *Rhodotorula mucilaginosa*, *Rhodotorula toruloides* CBS14 (Synonym: *Rhodosporidium toruloides*)*, Trichosporon asahii*, and *Cutaneotrichosporon oleaginosus* were compared in terms of growth and lipid accumulation, as well as fatty acid profiles. All strains are fromm Basidiomycota phylum. *R. glutinis*, *R. mucilaginosa*, and *R. toruloides* are close strains from genera *Rhodotorula* (class Microbotryomycetes, order Sporidiobolales). Lipid accumulation for these strains on glucose can reach 72, 15, and 58% (w/w) on a dry weight basis, respectively [22, 23]. In addition to lipid accumulation, these three yeast strains have attracted attention due to their natural ability to produce carotenoids, both being industrially relevant compounds [24, 25]. Furthermore, they can grow on a wide range of carbon sources [25–27]. For example, *R. mucilaginosa* has been grown on sugarcane bagasse, wheat straw, and wheat bran hydrolysate as well as Durian peel hydrolysates [22]. It is worth mentioning that the improvements in the genetic engineering tools and multi-omics data availability of *R. toruloides* have led to increased interest and application in both academy and industry [25, 28]. *T. asahii* and *C. oleaginosus* are from genera *Cutaneotrichosporon* and *Trichosporon* (class Tremellomycetes, order Trichosporonales), which were reported to be close relatives in this class [29]. Lipid accumulation for these strains on glucose has reached 33% and 53% on a dry weigh basis [30, 31]. *C. oleaginosus* yeast can grow and accumulate lipids up to 63.2%, 45%, 39.6%, 69.5% on a wide range of substrates, including volatile fatty acids, seagrass waste hydrolysate, waste-activated sludge, and aromatics, respectively [32–34]. *Cutaneotrichosporon oleaginosus* is a promising organism in this regard as a potential source of fatty alcohols and TAGs and lipids from *Cutaneotrichosporon oleaginosus*, resembles a cocoa butter-like fatty acid composition [35]. In a recent study, *C. oleaginosus* yielded the highest intracellular lipid amongst oleaginous yeasts through a new process by converting acetic acid and sugar into lipid [11]. Strains were selected that showed process flexibility and robustness in terms of sugar utilization and tolerance to fermentation inhibitors [36].

For correlative reference, we initially carried out comparative growth experiments for the strains on four monomeric sugars, separately: glucose, xylose, mannitol, and N-acetylglucosamine. Next, the strains were cultivated and analyzed on complex biomass hydrolysates derived from terrestrial and marine biomass. In the fermentations performed, different substrate preferences and associated growth efficiencies of the selected yeasts could be determined. Finding the yeast strains which are capable of tolerating the by-products of hydrolysis and pre-treatment processes that potentially have inhibitory effects is an essential step for the industrialization of yeast oil production using complex biomass hydrolysates as a fermentation feedstock [27].

To diversify the application of yeast oils, modulation of the fatty acid distribution of generated triglyceride oils is required. This can be achieved by altering the fermentation conditions [37]. In our study, we demonstrate that metabolizing different carbon sources can significantly change the fatty acid distributions. This is the first systematic study comparing the sugar utilization and inhibitory effects of the hydrolysates for an array of well-described oleaginous yeasts using both defined and complex fermentation media.

## Results

### Sugar uptake, and growth efficiencies

**Fig. 1:**
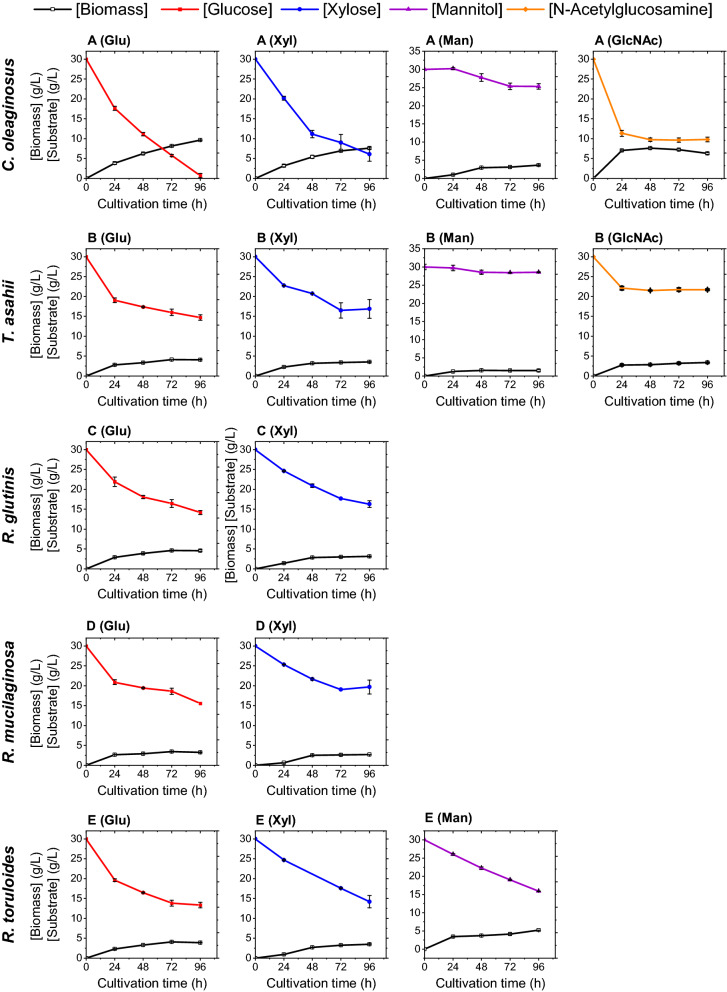
Growth rates and substrate consumption of each yeast strain in the synthetic media: **A** *C. oleaginosus* growth rates and substrate consumption on each monomeric sugar as the only carbon source; **B** *T. asahii*. **C** *R. glutinis*. **D** *R. mucilaginosa*. **E** *R. toruloides*

*Synthetic media containing sole carbon sources* Four different monomeric sugars, including glucose, xylose, mannitol, and N-acetylglucosamine, were used as sole carbon source in the cultivation media of all five investigated yeast strains (Table 1). The choice of sugars in synthetic media was based on the monomeric content of the hydrolysates tested in this work. In order to induce lipogenesis, nutrient limitations were applied in each medium: nitrogen limitation in media containing glucose (MNM-Glu), xylose (MNM-Xyl), and mannitol (MNM-Man), and phosphate limitation in the medium containing N-acetylglucosamine (MPM-GlcNAc). *C. oleaginosus* and *T. asahii* were able to metabolize all four types of monosaccharides, while *R. glutinis* and *R. mucilaginosa* did not grow in the MPM-GlcNAc and MNM-Man, and *R. toruloides* did not show growth in MPM-GlcNAc (Additional file 1: Fig. S1 compares the growth of all strains in each medium). In the synthetic media containing glucose, xylose, and N-acetylglucosamine, the maximum dry cell weight (DCW g_dried biomass_/L_culture_) was reached by *C. oleaginosus* (p-value ≤ 0.05), while in the synthetic media containing mannitol the maximum DCW was reached by *R. toruloides* (p-value ≤ 0.05). The data showed that the final DCW in the MNM-Glu was slightly higher than in the MNM-Xyl. The determined sugar uptake rates in each cultivation are in line with the DCWs (Fig. 1). In the synthetic minimal nitrogen media containing glucose (MNM-Glu), the sugar content was exhausted by *C. oleaginosus* during the cultivation in the MNM-Glu, resulting in the maximum biomass concentration (9.6 ± 0.1 g/L after 96 h of cultivation), while in the medium containing xylose (MNM-Xyl) 80% (w/w) of the available sugar was consumed. However, the other strains utilized glucose and xylose up to 55% (w/w) (Fig. 1 and Table 2). *C. oleaginosus* metabolized 66% (w/w) (20 g/L) of available GlcNAc, while *T. asahii* metabolized 30% (w/w) (9 g/L) GlcNAc over the cultivation period. Notably, by utilizing GlcNAc, *C. oleaginosus* reached its maximum DCW (7.61 ± 0.17 g/L) within 48 h, whereas the same biomass concentration in the MNM-Xyl was measured after 96 h (7.65 ± 0.25 g/L) (Fig. 1). In the medium containing mannitol, *R. toruloides* was the most efficient yeast strain amongst all tested in terms of sugar consumption (46% w/w of the available sugar).

**Table 1:** Sugar content of the synthetic media and complex hydrolysates

	Media	[Glucose] g/L	[Xylose] g/L	[Mannitol] g/L	[GlcNAc] g/L	[CSL] g/L
Synthetic media	MNM-Glu	30	–	–	–	–
	MNM-Xyl	–	30	–	–	–
	MNM-Man	–	–	30	–	–
	MPM-GlcNAc	–	–	–	30	–
Complex hydrolysates	*Laminaria digitata* hydrolysate	19.0 ± 0.1	4.4 ± 0.02	9.2 ± 0.04	nd	–
	Wheat straw hydrolysate	27.5 ± 0.3	14.0 ± 0.2	nd	nd	–
	Wheat straw hydrolysate + corn steep liquor	27.5 ± 0.3	14.0 ± 0.2	nd	nd	5.0

*nd* not detected

**Table 2:** Measurement of lipid productivity and yield, biomass yield, and total utilized sugar in all media

Strain	Media	Incubation time (h)	Lipid productivity (g/Lh)	Lipid yield g_Lipid_/g_Sugar_	Biomass yield g_Biomass_/g_Sugar_	[Total utilized sugar] g/L	Total sugar consumption % (w/w)
*C. oleaginosus*	MNM-Glu	72	0.066	0.19	0.337	24.2	80.8
		96	0.062	0.17	0.329	29.3	97.6
	MNM-Xyl	72	0.057	0.20	0.326	21.0	70.0
		96	0.036	0.15	0.306	23.9	79.7
	MNM-Man	72	0.011	0.177	0.679	4.7	15.5
		96	0.009	0.183	0.770	4.7	15.7
	MPM-GlcNAc	48	0.042	0.10	0.376	20.2	67.5
		72	0.027	0.10	0.357	20.4	67.8
		96	0.018	0.09	0.312	20.2	67.3
	BAH	72	0.045	0.13	0.675	24.6	73.8
		96	0.040	0.16	0.697	24.4	73.2
	WSH	72	0.091	0.18	0.421	34.9	83.2
		96	0.079	0.19	0.394	40.3	96.0
	WSH + CSL	72	0.097	0.19	0.373	40.2	95.8
		96	0.072	0.17	0.419	40.2	95.8
*T. asahii*	MNM-Glu	72	0.005	0.03	0.295	14.0	46.6
		96	0.011	0.07	0.269	15.3	51.0
	MNM-Xyl	72	0.005	0.03	0.258	13.5	45.0
		96	0.009	0.06	0.277	13.1	43.8
	MNM-Man	72	0.003	0.14	0.939	1.6	5.3
		96	0.001	0.07	1.058	1.4	4.8
	MPM-GlcNAc	48	0.004	0.02	0.333	8.5	27.6
		72	0.003	0.03	0.389	8.3	27.6
		96	0.003	0.03	0.407	8.3	27.6
	BAH	72	0.020	0.08	0.780	18.1	54.5
		96	0.019	0.10	0.804	18.5	55.5
	WSH	72	0.007	0.04	0.401	13.1	31.3
		96	0.009	0.06	0.460	13.8	32.8
	WSH + CSL	72	0.008	0.03	0.266	18.5	44.0
		96	0.009	0.04	0.239	23.6	56.2
*R. glutinis*	MNM-Glu	72	0.009	0.05	0.341	13.5	45.2
		96	0.011	0.06	0.291	15.8	61.8
	MNM-Xyl	72	0.012	0.07	0.243	7.3	41.1
		96	0.007	0.05	0.230	13.7	45.7
	BAH	72	0.035	0.11	0.713	23.3	69.9
		96	0.028	0.12	0.723	23.5	70.5
	WSH	72	0.020	0.08	0.549	18.7	44.5
		96	0.019	0.09	0.488	19.7	46.8
	WSH + CSL	72	0.027	0.08	0.427	23.9	57.0
		96	0.023	0.08	0.449	27.0	64.3
*R. mucilaginosa*	MNM-Glu	72	0.004	0.03	0.303	11.4	37.9
		96	0.007	0.04	0.225	14.4	48.1
	MNM-Xyl	72	0.005	0.03	0.246	11.0	36.6
		96	0.003	0.03	0.268	10.3	34.4
	BAH	72	0.021	0.07	0.704	20.6	62.0
		96	0.014	0.06	0.709	20.6	62.0
	WSH	72	0.010	0.05	0.553	13.3	31.6
		96	0.007	0.05	0.518	14.1	33.7
	WSH + CSL	72	0.010	0.04	0.468	19.2	45.7
		96	0.009	0.04	0.474	21.5	51.2
*R. toruloides*	MNM-Glu	72	0.019	0.09	0.253	16.2	53.9
		96	0.018	0.10	0.242	16.7	55.6
	MNM-Xyl	72	0.013	0.07	0.260	12.4	41.4
		96	0.011	0.07	0.219	15.8	52.7
	MNM-Man	72	0.022	0.14	0.375	11.0	36.6
		96	0.021	0.14	0.367	14.1	47.0
	BAH	72	0.038	0.11	0.614	25.6	76.8
		96	0.015	0.05	0.593	26.3	78.9

The stationary phases were determined according to the DCWs, and afterward time points 72 h and 96 h were selected to analyze the lipid accumulations

*Complex biomass hydrolysates* To determine the growth of the yeasts on terrestrial lignocellulosic and marine biomass hydrolysates, we chose two complex media for further analysis; brown algae hydrolysate (BAH) from *Laminaria digitata* and wheat straw hydrolysate (WSH). Additionally, the wheat straw hydrolysate was then supplemented with corn steep liquor as a nitrogen source (WSH + CSL) (Table 1). The biomass yield per gram substrate consumed in Table 2 indicates that, relative to synthetic media, cultivations in the hydrolysates enhanced the growth efficiency (g_biomass_/g_utilized sugar_) of all yeast strains considerably (p-value ≤ 0.05).

**Fig. 2:**
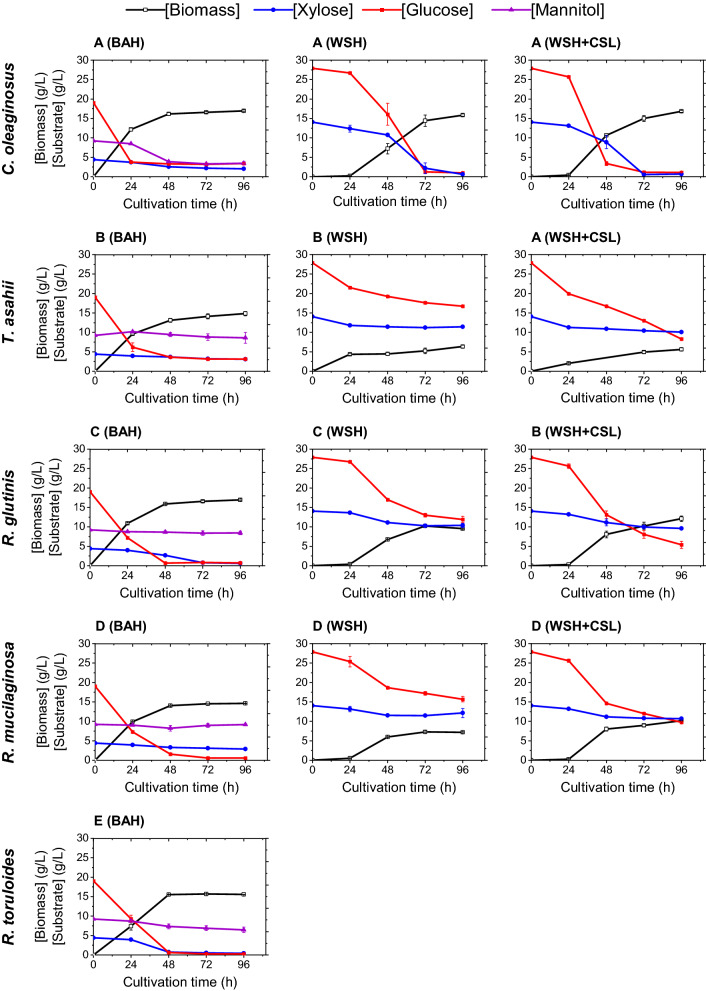
Growth rates and substrate consumption of each yeast strain in the complex media: **A** *C. oleaginosus* growth rates and substrate consumption **B** *T. asahii*. **C** *R. glutinis*. **D** *R. mucilaginosa*. **E** *R. toruloides*

The BAH was composed of three carbon sources: glucose, xylose, and mannitol, and it allowed the growth of all five yeast strains (Table 1). In particular, *C. oleaginosus* and *R. toruloides* utilized all three sugars simultaneously. In addition, an improved mannitol consumption rate by *C. oleaginosus* could be determined. Over 80% (w/w) of the glucose content, 54% (w/w) xylose, and 62% (w/w) mannitol were consumed by *C. oleaginosus*, and over 90% of xylose and glucose, and 30% (w/w) of mannitol were consumed by *R. toruloides* (Fig. 2 and Additional file 2: Table S1). An efficient co-utilization of glucose and xylose (97% and 86% w/w, respectively) was seen in *R. glutinis* as well. An uptake of less than 10% (w/w) of the mannitol source could be determined in *R. glutinis.* Interestingly, the growth trend, the final DCW, and the biomass yield of *R. glutinis* matched those of *C. oleaginosus*, both showing the highest biomass growth in the BAH (DCW after 96 h; 16.95 ± 0.21 g/L and 16.97 ± 0.25 g/L, corresponding to 0.72 g_biomass_/g_sugar_ and 0.69 g_biomass_/g_sugar_, respectively (Table 2). Moreover, *T. asahii* and *R. mucilaginosa* consumed glucose preferentially to xylose and mannitol, indicating the glucose carbon catabolite repression. Even though *T. asahii* is taxonomically closer than *R.* *glutinis* to *C. oleaginosus*, it did not perform well in the BAH in the current study [29].

**Fig. 3:**
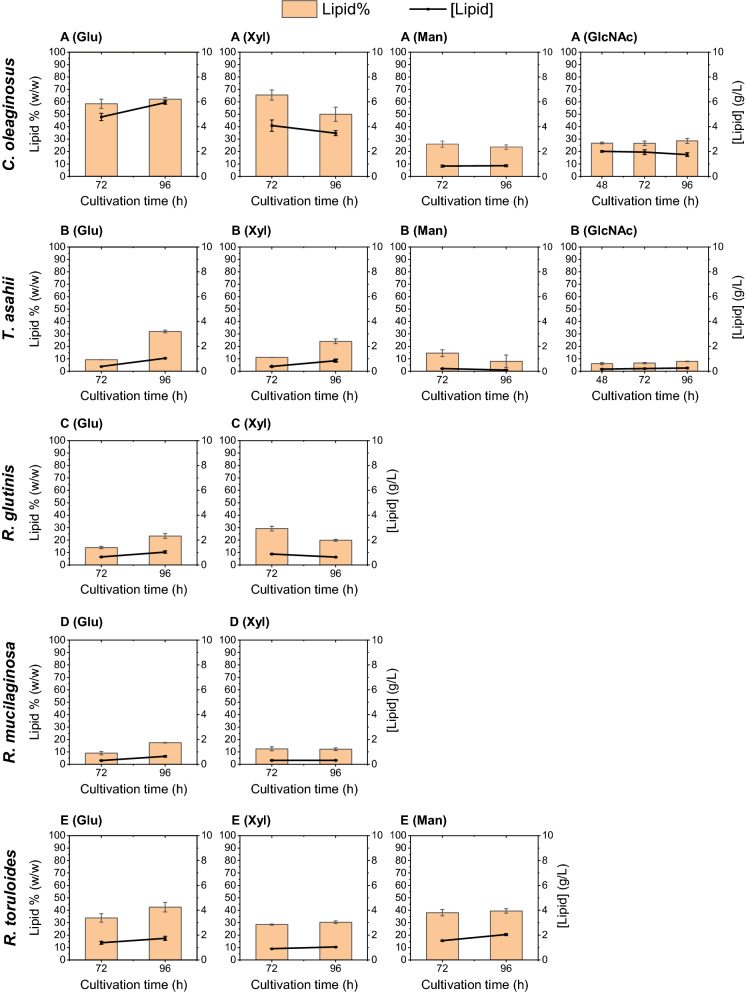
Lipid contents and total lipid of each yeast strain in the synthetic media: **A** *C. oleaginosus* lipid content and lipid concentrations in synthetic medium containing each monomeric sugar as the only carbon source; **B** *T. asahii*. **C** *R. glutinis*. **D** *R. mucilaginosa*. **E** *R. toruloides*

The WSH contained glucose and xylose (Table 1) and our data demonstrated that all strains tested are able to utilize both monosaccharides as sole carbon sources in the synthetic media, resulting in biomass and lipid formation (Fig. 3). However, *R. toruloides* did not exhibit any growth when cultivated in the WSH. In contrast, the final biomass accumulation, and consumed sugar of *C. oleaginosus* in this hydrolysate (sugar exhausted, DCW at 15.86 ± 0.28 g/L) was remarkably higher compared to the other strains. Other yeast strains utilized only up to 50% of available, fermentable sugars (Fig. 2). In general, in BAH, better performance in terms of cell mass production by *R. glutinis*, *T. asahii*, *R. mucilaginosa* and *R. toruloides* could be observed when compared to WSH (Fig. 2).

Therefore, CSL was added to WSH as a source of nutrients to boost growth. Adding CSL to the WSH led to improvements in the DCW for *C. oleaginosus*, *R. glutinis* and *R. mucilaginosa* by 6%, 24%, and 41% (w/w) at the end of the cultivation time, respectively. This enhancement of growth was mirrored in the sugar consumption rates. *R. glutinis,* and *R. mucilaginosa* increased their monosaccharide uptake in the wheat straw hydrolysate supplied with CSL, compared to WSH with no extra nitrogen source (Table 2). *T. asahii* consumed more sugar in the presence of CSL as well, however, no difference in its biomass and lipid yield could be measured due to cell-aggregation of *T. asahii* in the WSH cultivation after 72 h.

### Lipid yield and productivity

*Synthetic media containing sole carbon sources* The total intracellular lipid contents (gram lipid per gram biomass) varied with the examined yeast strains and the choice of culture medium. Except for MNM-Man, *C. oleaginosus* demonstrated the highest lipid contents in the tested media (Fig. 3). In MNM-Man, *R. toruloides* yielded the highest lipid content (39 ± 1.7% w/w of dry weight in 96 h). This yeast strain achieved a higher lipid content in MNM-Glu (42.4 ± 3.8% (w/w) of dry weight in 96 h) compared to MNM-Xyl (30.4 ± 0.9% w/w in 96 h). Notably, *R. mucilaginosa* and *T. asahii* also showed better performance on glucose than on xylose. The lipid content in *T. asahii* reached its maximum after 96 h cultivation in both MNM-Glu and MNM-Xyl, measuring at 31% (w/w) and 24% (w/w) on a dry weight basis, respectively. By contrast, cultivation of *C. oleaginosus* in xylose-containing synthetic medium led to an accelerated, and higher lipid accumulation (65% w/w within 72 h), compared to glucose medium (58% and 62% w/w in 72 h and 96 h, respectively). This is consistent with previous literature data, which showed that *C. oleaginosus* yielded a higher lipid content in medium containing xylose (57% w/w in 72 h), compared to medium containing glucose (48% w/w in 72 h) [35]. However, it is notable that 20% (w/w) of the xylose remained unconsumed, while glucose was metabolized quantitatively. In this study, the same behavior was observed in *R. glutinis* where it reached a higher lipid content in a shorter time by utilizing xylose in comparison to glucose (29 ± 1.8% w/w with a substrate to lipid conversion rate of 7 mg_lipid_/g_sugar_ in 72 h in MNM-Xyl and, 23 ± 1.9% w/w with 6 mg_lipid_/g_sugar_ in 96 h in MNM-Glu, Table 2). In the MPM-GlcNAc *T. asahii* and *C. oleaginosus* were the only strains that could utilize the N-acetyl glucosamine, the latter was able to accumulate 28 ± 2.1% of its biomass as lipid in this medium, while *T. asahii* did not produce high amounts of oil under these conditions (7.9 ± 0.2% w/w). In addition, our data indicated that the lipid content obtained by *C. oleaginosus* did not increase significantly between the time points of 48 h and 96 h of cultivation in MPM-GlcNAc (p-value > 0.05).

**Fig. 4:**
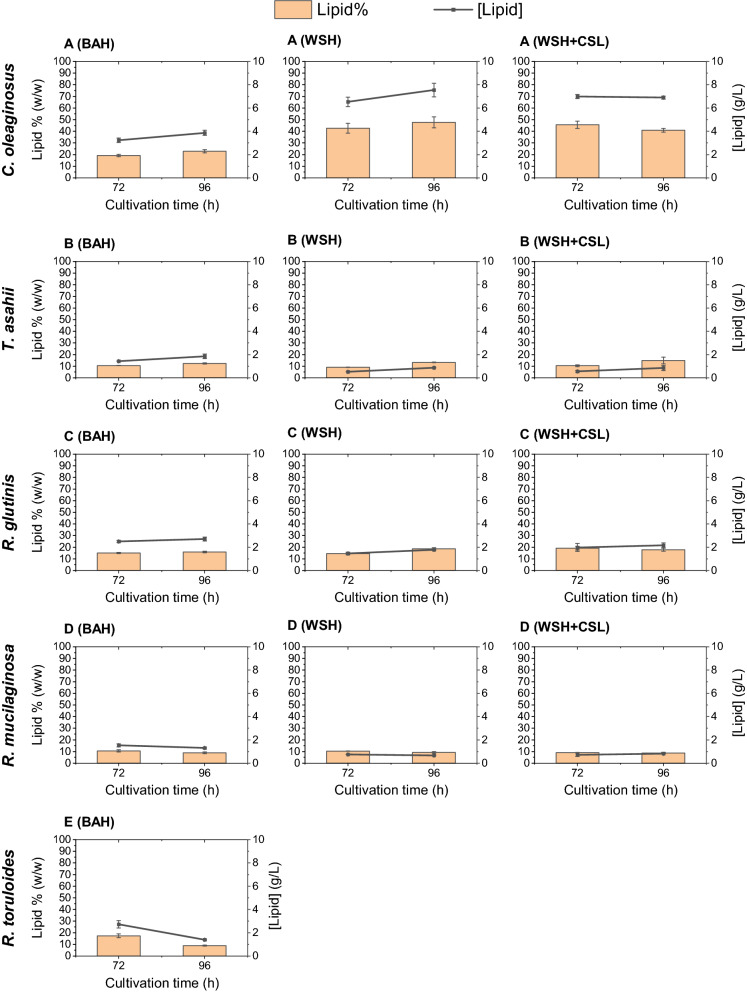
Lipid contents and total lipid of each yeast strain in the complex media: **A** *C. oleaginosus* lipid content and lipid concentrations in each complex medium **B** *T. asahii*. **C** *R. glutinis*. **D** *R. mucilaginosa*. **E** *R. toruloides*

*Complex biomass hydrolysates* The final total lipid (g/l) were generally elevated on the complex carbon sources (Fig. 4), compared to synthetic media containing sole carbon sources (Fig. 3). For *C. oleaginosus*, *R. glutinis*, and *T. asahii*, the lipid contents were higher in WSH than in BAH (Fig. 4). *C. oleaginosus* built up biomass equally in the WSH and BAH (Fig. 2). However, cultivation in WSH yielded higher lipid productivity than BAH after 96 h, providing 0.078 g/Lh (15.86 g/L biomass, 47% w/w lipid) and 0.04 g/Lh (16.9 g/L biomass, 22% w/w lipid), respectively (Table 2). The concentration of fermentable sugars in WSH was higher compared to BAH. Notably, all available sugars were exhausted in the WSH, and the final lipid yield (per carbon unit consumed) was higher in WSH cultivations (Table 2). *C. oleaginosus* accumulated 0.19 g lipid per gram of carbon substrate consumed in WSH within 96 h, which is higher than that of BAH (0.16 g_Lipid_/g_Sugar_). This correlation in MNM-Glu and MNM-Xyl was calculated at 0.17 and 0.2 g_Lipid_/g_Sugar_ after 96 h and 72 h, at the time point where the maximum lipid content was reached, respectively. The other strains, however, performed best on BAH, which resulted in a higher total lipid and productivity compared to WSH (Table 2). Exemplary, *R. glutinis* had lipid productivity of 0.028 g/Lh (16.95 g/L biomass, 15% w/w lipid) in BAH while this value in WSH was 0.019 g/Lh (9.5 g/L biomass, 18% w/w lipid). Adding CSL as a nitrogen source to WSH did not show any negative impact on the maximum lipid yield per gram sugar consumed (g_Lipid_/g_Sugar_) (Table 2). Furthermore, the addition of CSL to WSH did not improve the lipid content (% w/w on a dry weight basis) of *C. oleaginosus* and *R. glutinis* but the maximum lipid content in WSH + CSL was achieved in a shorter time (72 h), resulting in higher lipid productivity (Table 2). In the complex BAH, *R. toruloides* performed as well as *R. glutinis* in terms of total lipid and biomass formation (Figs. 2, 4).

### Fatty acid profile variations associated with media choice and composition

**Fig. 5:**
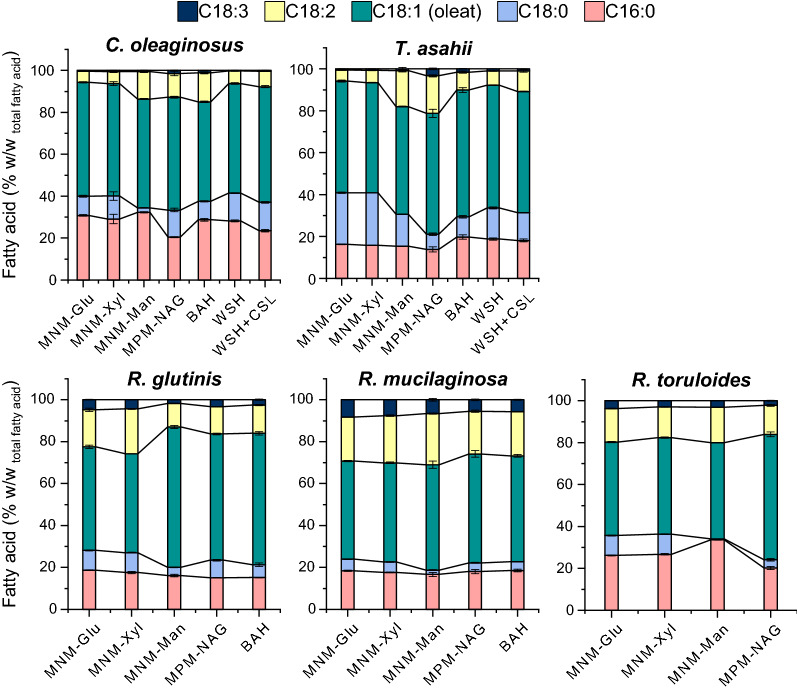
Fatty acid profile of each strain in different media after 96 h incubation

*Synthetic media containing sole carbon sources* An overview of the fatty acid profiles is summarized in Fig. 5 (and Additional file 2: Table S2). In general, the fatty acid profiles showed variations in different media and strains, while oleic acid (C18:1) remained the predominant fatty acid throughout all tested strains. The cultivations in the two different MNM, supplemented with xylose or glucose resulted in comparable fatty acid profiles for each strain. In these synthetic media, *T. asahii* reached the highest percentage of Stearic acid C18:0 amongst all strains analyzed (25% w/w_total fatty acid_) which was decreased significantly in MPM-GlcNAc (p-value ≤ 0.05). *T. asahii* and *C. oleaginosus* were the only strains that exhibited growth on N-acetylglucosamine. Notably, the resulting fatty acid profile depicted a higher level of unsaturated FAs in this medium when compared to synthetic Glu/Xyl-containing media. To that end, *C. oleaginosus* synthesized twice the concentration of C18:2, and a lower ratio of C16:0 in MPM-GlcNAc. Interestingly, MNM-Man resulted in an increase in unsaturated fatty acids of *C. oleaginosus* and *T. asahii* as well, while the C18:0 percentages were lower in this media (p-value ≤ 0.05). This fatty acid was measured lower than 1% in MNM-Man in *R. toruloides*.

*Complex biomass hydrolysates* The cultivation of all strains in BAH could increase the total unsaturated fatty acids contents. *C. oleaginosus* showed a higher percentage of C18:2 resulting in a decrease of oleic acid and a final increase of PUFAs by 8% (w/w_total FAs_). While all strains yielded lower saturated FAs and in turn higher unsaturated FAs, in BAH. In addition, *R. toruloides* and *R. glutinis* had a notable surge in the C18:1 concentration when cultivated in BAH. *T. asahii* and *R. mucilaginosa* in general produced a higher amount of unsaturated FAs. *R. mucilaginosa* reached the highest percentage of unsaturated FAs (81% w/w_total FAs_) as well as PUFAs (30% w/w_total FAs_) amongst all strains tested in BAH. Cultivations in WSH (± CSL) had slighter effects on the fatty acid profiles. By comparison to the synthetic media, this hydrolysate resulted in a 10% (w/w_total FAs_) and 7% (w/w_total FAs_) increase in the C18:1 ratio of *R. glutinis*, and unsaturated FAs obtained from *T. asahii,* respectively.

## Discussion

Five well-established oleaginous yeast strains were studied for their carbon source preference, fermentation inhibitor tolerance, and lipid yields on complex biomass hydrolysates derived from wheat straw and brown algae (*Laminaria digitate*). For control and calibration purposes strains were also cultivated in defined, synthetic minimal media containing the main component sugars of both complex media. Moreover, the yeast's capacity to metabolize N-acetylglucosamine, the monomeric component of chitin, which is the second most abundant biopolymer after cellulose in nature, was examined systematically for the first time. Additionally, the use of corn steep liquor as a cost-efficient nitrogen source was examined in combination with complex biomass hydrolysates. All strains tested in this study were able to channel the extra-cellular carbon towards lipid neo-synthesis under nutrient limiting conditions (nitrogen limitation in MNM-Glu, MNM-Xyl, and MNM-Man, and phosphate limitation in MPM- GlcNAc) which indicates the lipid accumulation through the “de novo” lipid biosynthesis pathway [7, 29, 38, 39]. In this study, we utilized the two-phase cultivation protocol involving an initial growth period, followed by nutrient limitation in each defined synthetic media [40].

The growing interest in YOs during the past decades shows that YO will not only remain a significant part of the biochemical research but also turn into a bigger platform in the respective industries. In order to fully recover the resources, it is preferred that the oleaginous strain consumes all types of carbon substrates in the hydrolysate. For the feasibility of such a process, it is necessary to identify the yeast strains that can tolerate the hydrolysis by-products of the local abundant feedstocks. Several studies investigated *Laminaria* hydrolysates for lipid accumulation in different yeasts. The wheat straw hydrolysate was investigated as well for microbial oil production [33, 41, 42]. This is the first systematic study comparing differential substrate preferences, growth efficiencies as well as tolerance against the potential inhibitory effects of the hydrolysates from both terrestrial and marine origin, as well as their respective synthetic control medium, for an described prominent oleaginous yeasts.

A number of previous studies have investigated oleaginous yeasts in terms of utilization of pentose sugars, while this study represents the first comparative investigation on growth and lipid production using a pentose sugar both as a sole carbon source and as a part of a complex hydrolysate. Pentose sugars like xylose, generated through the hydrolysis of the lignocellulosic material, are challenging substrates since most microorganisms cannot utilize them [43]. In a study, it has been shown that most of the strains of *Yarrowia* clade were able to utilize xylose in switchgrass hydrolysate [44]. Furthermore, lignin makes up 10–30% of the lignocellulosic biomass by weight [45] and is depolymerized by the pre-treatment of wheat straw. Its degradation products such as vanillin, 4-hydroxybenzaldehyde, and syringaldehyde are potential growth inhibitors for most microorganisms. *C. oleaginosus* is reported to have a high tolerance towards a wide range of lignin-derived aromatic compounds (e.g., resorcinol and protocatechuate) as well as acetic acid, the by-products of wheat straw hydrolysis. In fact, *C. oleaginosus* can utilize these harmful compounds as substrates to accumulate lipids [11, 34]. In the wheat straw hydrolysate, glucose and xylose are co-utilized by *C. oleaginosus*. In a previous study, it is reported that a co-utilization of glucose and xylose by *C. oleaginosus* in a defined synthetic media (glu:xyl mass ratio at 45:25 g/g equal to 1.8:1 g/g) resulted in the substrate to lipid conversion of 16.4% g_lipid_/g_sugar_ [46]. By comparison, a higher conversion rate (18.7% g_lipid_/g_sugar_) was achieved in the current study, when this strain was cultivated in the WSH (with a similar glu:xyl mass ratio at 27:14 g/g equal to 1.9:1 g/g). The higher rate in *C. oleaginosus* in our study can be explained by its potential for utilization of lignin-derived aromatic compounds [34]. This data demonstrates that this strain is capable of co-utilizing the aromatics and both glucose and xylose towards lipid production. According to our data, the highest growth on WSH was achieved by *C.* *oleaginosus*. The WSH used in our study was not detoxified. Therefore, it could be concluded that the other strains examined in this study are not as tolerant to the phenolic compounds derived from lignin hydrolysis or other growth inhibitors resulting from pretreatment steps as *C. oleaginosus*. *R. toruloides* CBS14 performed as second-best in terms of growth as well as lipid on glucose and xylose in synthetic media (Fig. 3, and Additional file 1: Fig. S1). However, it did not exhibit any growth in WSH, indicating that the hydrolysate components inhibited the growth. Similar behavior of *R. toruloides* was reported by Yu et al., whereby, unlike the non-detoxified hydrolysate, the detoxified WSH allowed growth and lipid accumulation. Furthermore, the types of growth inhibitors generated by mild acidic pretreatment of WSH were identified [33]. By comparison, *Y. lipolytica* accumulated lipid up to 4.6% of its dry cell weight in this hydrolysate [33]. The tolerance of yeast strains to these inhibitors could be improved using genetic engineering tools. Further research is needed to determine the mechanisms underlying the different tolerance degrees of yeast strains to the inhibitors by metabolome analysis.

Interestingly, the addition of CSL, a rich source of nitrogen and nutrients, to the WSH enhanced the growth and accelerated the lipid accumulations of all strains tested. Although the limited growth of *T. asahii*, *R. glutinis*, *R. mucilaginosa*, and *R. toruloides* was thought to be due to their lower tolerance to the lignin-derived aromatics, it appeared that their performance was improved by adding CSL as a source of nitrogen and nutrients. *C. oleaginous* was able to consume all available sugars in the WSH (42 g/L sugar in 96 h). Therefore the addition of CSL did not affect its final yield in comparison to the other strains in WSH + CSL *C. oleaginosus* remained oleaginous despite a higher nitrogen concentration. This study demonstrates that cultivation with the wheat straw hydrolysate resulted in 7.5 g/L lipids, 47% of its dry weight by *C. oleaginosus*. A continuous or fed-batch fermentation could result in even a higher final lipid and biomass, where the required carbon source is provided in the presence of CSL.

According to Papanikolaou et al. as far as glucose metabolism is concerned, 100 g of glucose (equal to 0.56 mol) could yield 1.1 mol of Acetyl-CoA. Xylose however can be catabolized by the phosphoketolase reaction as well as the pentose phosphate pathway, resulting in 1.2 mol and 1.0 mol Acetyl-CoA from 100 g xylose (0.66 mol), respectively. Hence, under lipid accumulating conditions, where all the Acetyl-CoA is utilized to form lipids, the theoretical cellular lipid yield is 0.32 g per gram consumed glucose. When xylose is utilized under the involvement of the phosphoketolase pathway, the value slightly increases to 0.34 g/g [39]. The maximum carbon:carbon efficiency for *C. oleaginosus* and *R. glutinis* in MNM-Xyl was also calculated to be higher than that in MNM-Glu (20 mg_lipid_/g_sugar_ and 17 mg_lipid_/g_sugar_ for *C. oleaginosus* and 7 mg_lipid_/g_sugar_ and 6 mg_lipid_/g_sugar_ for *R. glutinis* at their highest lipid accumulation points, respectively (Table 2). Based on the stoichiometry of sugar metabolism, these findings could suggest, that *C. oleaginosus* and *R. glutinis* use the phosphoketolase pathway for the xylose metabolism, under the evaluated cultivation conditions. Phosphoketolase(s) have been found formerly in *R. glutinis* and *R. toruloides* as well [47, 48]. The exhaustion of xylose by these two strains as well as their higher carbon:carbon efficiencies in the BAH by comparison to synthetic media (biomass and lipid yields reported in Table 2) could be an indication of a relatively higher phosphoketolase activity under the tested conditions in comparison to the other strains. The better performance of *C. oleaginosus* on WSH than BAH in terms of both lipid yield per gram sugar consumed and lipid content can again be explained by the involvement of the phosphoketolase pathway. Especially when considering the fact that the pentose content is much higher in the WSH in contrast to the BAH. A further investigation is needed to pinpoint the pathway preferences in oleaginous yeast, especially the effect of the C/N ratio and co-consumption of different sugars on the sugar assimilation pathways.

In addition to glucose and xylose, BAH contains mannitol. This sugar alcohol is one of the major carbon sources of brown algae. Co-utilization of all three sugars was observed for *R. toruloides* and *C. oleaginosus.* Until now, there are only a few studies reporting yeast strains that are naturally able to absorb mannitol and channel it into lipids biosynthesis [49]. The mannitol uptake mechanism, as well as the consumption effect of mannitol on fatty acid profiles and lipid accumulation in oleaginous yeasts, are not yet well studied. It was shown that in an engineered *S. cerevisiae*, the mannitol assimilation is conducted through mannitol transporters into the cytoplasmic space and subsequently metabolized to D-fructose by mannitol-2-dehydrogenase consuming NAD^+^ [14, 50]. Interestingly, a study on the marine yeast *Rhodosporidiobolus fluvialis* Y2 demonstrated that mannitol uptake resulted in higher levels of PUFAs, suggesting the NADH required for desaturases is provided by mannitol-2-dehydrogenase, therefore the fatty acid desaturases become more active in presence of mannitol [49]. A similar effect was seen in our study. As a result of mannitol uptake as a sole carbon source in the MNM-Man, the amount of PUFAs (C18:2) produced by *C. oleaginosus* and *T. asahii* was increased and a considerable decrease in C18:0 was determined, which could demonstrate a mannitol-dependent activation of Δ12 and Δ9 desaturases. These changes were also detected in the BAH in the presence of mannitol, nevertheless, *T. asahii* also showed a higher amount of C18:1 in BAH which could be due to a higher Δ9 desaturase activity and slightly lower Δ12 desaturase activity when utilizing a mixture of monomers compared with utilizing mannitol as a sole carbon source. In contrast, in *R. toruloides*, the mannitol utilization resulted in an elevated level of palmitic acid (C16:0).

When using N-acetylglucosamine as a sole carbon source, only *C. oleaginous* and *T. asahii*, were able to utilize this sugar. This observation is consistent with genome and transcriptome analysis of *C. oleaginosus* which predicted the genes involved in GlcNAc utilization including GlcNAc kinase with homology to the *C. albicans* NAG kinase, as well as N-acetyl-glucosamine-6-phosphate deacetylase [Triol1|281629] and glucosamine-6-phosphate deaminase [Triol1|281628]) [29]. Notably, *C. oleaginosus* was far more efficient than its closest sequenced relative, *T. asahii*, in terms of GlcNAc uptake as well as growth and lipid accumulation in this media. The metabolism of GlcNAc is not well characterized in the oleaginous yeasts. An N-acetylglucosamine kinase (Hxk1) in *C. albicans* was first reported in 1974 [51]. It has been shown that the GlcNAc uptake capacity of the cells is proportional to the level of a specific binding protein expressed by the cells [52]. In *C. albicans*, the N-acetylglucosamine transporter (Ngt1) mediates the entry of GlcNAc into the cells, which represented the first eukaryotic GlcNAc transporter. In addition, the *Saccharomyces cerevisiae* expressing Ngt1 exhibited GlcNAc uptake ability. This showed that the Ngt1 in a direct way functions as a GlcNAc transporter [53]. Inside the cells, the binding of free GlcNAc to the Ngs1 protein (a GlcNAc sensor and transducer) is required for GlcNAc signaling. It induces other transcriptional responses to this amino sugar in the nucleus, and it is conserved in various fungi. The mutations in the Ngs1, the binding site of N-acetylglucosamine, abolished growth on GlcNAc in *C. albicans*. [54, 55]. Until now, no transporter and sensor-transducer for GlcNAc is identified in *C. oleaginosus* or other oleaginous yeasts. However, the high efficiency of *C. oleaginosus* to utilize this sugar compared to *T. asahii* (63% w/w (19 g_GlcNAc_/ L) and 26% w/w (8 g_GlcNAc_/ L) uptake in 24 h) indicates a more efficient GlcNAc metabolism in this yeast, most likely including highly effective transporter and sensor-transducer. It is noteworthy that GlcNAc metabolism is reported to release NH4^+^ and acetate leading to a higher of extracellular pH [56, 57]. *C. oleaginosus* in general is able to consume and channel acetate to lipid biosynthesis [58]. However, the accumulation of ammonia and high pH could interfere with further growth due to their cytotoxicity effects [59]. The extreme changes of pH could be avoided under controlled conditions in a continuous or batch fermentation mode. Therefore, the tolerability of oleaginous yeasts to ammonia and acetate plays an important role in efficient lipid production from GlcNAc. In order to further identify the respective system in *C. oleaginosus*, proteomic analysis is required. A better valorization of chitin-based feedstocks can be accomplished through modification of yeast strains, therefore a better understanding of GlcNAc metabolism is necessary.

## Conclusion

Among the yeast strains investigated in this study, *C. oleaginosus* performed as the most versatile strain in terms of substrate utilization, productivity, and tolerance towards fermentation inhibitors in the complex media. It yielded 7.5 g/L lipids in the wheat straw hydrolysate. Commercially available lignocellulosic residues, like WSH, are currently mainly used for the production of bioethanol [60]. A better energetic and economic valorization of lignocellulosic waste could be accomplished by just switching this process to lipid production, as a product of higher energy density is formed. To that end, CSL was also shown as a promising inexpensive source of nitrogen to improve the growth to produce YOs. In the current study, CSL enhanced the growth in the oleaginous strains such as *R. glutinis*, *C. oleaginosus*, and *R. mucilaginosa*. In this work, *C. oleaginosus*, *R. glutinis*, and *R. toruloides* showed a high potential for lipid production on the marine biomass hydrolysate with efficient pentose utilization. Furthermore, this study has demonstrated that the fatty acid profile varies when cultivation is carried out on different feedstocks, especially on BAH due to the presence of mannitol. Combining the element of carbon source with other formerly-studied factors such as temperature [61–63], nitrogen source [31], and oxygen [37, 64] will enable us to achieve the desired fatty acid composition for diverse applications. Moreover, the degree of fatty acid saturation determines the physiochemical properties of resulting biofuels. These properties, which include Iodine Value (IV), Cetane Number (CN), Higher Heating Value (HHV), Kinematic Viscosity (KV), and Density, have been qualified for *C. oleaginosus*, *Y. lipolytica*, *R. turoloides*, and *L. starkeyi* and found to be comparable with palm oil. These oils are also positioned within internationally accepted biofuel standard limits for US biodiesel: ASTM D6751 and for EU biodiesel: EN 14214 [65–68]. Another viable application for *C. oleaginosus* targets the food sector. Solvent-free lipid extraction from *C. oleaginosus*, grown at a technical scale, in addition to life cycle analysis, showcased the economic feasibility of using this microbial oil in the food industry.

## Methods and materials

### Strains, environmental samples and media

Five prominent oleaginous yeast strains were screened in this study. *Cutaneotrichosporon oleaginosus* ATCC 20,509 (DSM-11815) was obtained from the Deutsche Sammlung von Mikroorganismen und Zellkulturen (DMSZ) (Braunschweig, Germany), and *Rhodosporidium toruloides* CBS 14 was obtained from Centraalbureau voor Schimmelcultures (CBS). *Rhodotorula glutinis* (IBY050), *Trichosporon asahii* (IBY051), and *Rhodotorula mucilaginosa* (IBY052) were obtained from the Werner Siemens-Chair of Synthetic Biotechnology (WSSB) culture collection. The strains were transferred and maintained on YPD- agar plates. The inoculum media was YPD as well. Generic synthetic media for lipid accumulation include nitrogen limitation medium (carbon source: glucose, xylose, or mannitol 30 g/L); and phosphate limitation medium (carbon source: N-acetyl glucosamine 30 g/L); [35]. The pH of all synthetic media was adjusted to 6.5 prior to sterilization. The complex medium used in this screening were wheat straw hydrolysate, corn steep liquor, and hydrolysate of brown algae *Laminaria digitata*. The brown algae were hydrolyzed enzymatically at a 2 L scale according to our previous study [10]. The wheat straw hydrolysate was obtained from Clariant (Germany). The hydrolysate was diluted and subjected to crossflow filtration (10 kDa polyethersulfone filter; Pall Corporation, US) to remove all proteins. The pH of both hydrolysates was set at 6.5 prior to sterile filtration. The corn steep liquor was commercially available and obtained from the TT baits (Germany) in a powder form. It was autoclaved at 134 °C for 20 min and added to WSH at the final concentration of 5 g/L under sterile conditions before the inoculation.

### Cultivation conditions

Single colonies of the yeast strains from YPD-agar plates were inoculated into 20 mL of YPD medium overnight as the pre-cultures. The cultivations were started by inoculating 100 mL of each medium to an OD_600 nm_ of 0.1 in a 500 mL baffled shaking flask. All experiments were done in triplicates. The shaking flasks were incubated at 28 °C and 120 rpm for 96 h. Samples were taken each 24 h and stored at – 20 °C.

### Fatty acid determination

Two ml cultivation medium was pelleted, washed with ddH_2_O, and lyophilized. Lyophilization was carried out for 2 days at − 80 °C and 0.04 mbar (VaCo 5, Zirbus Technology, Germany). Fatty acid analysis was done according to the modified protocol of Griffiths et al. [69]. Glyceryl trinonadecanoate (C19:0-TAG, 0.2 mg/mL in GC grade toluol) was added prior to the reaction as an internal standard. The dried biomass was directly converted into fatty acid methyl esters (FAME) by simultaneous extraction and transesterification of yeast lipids using 0.5 M Sodium methoxide solution in GC grade methanol and hydrogen chloride-methanol solution (Sigma, Germany) the fatty acid profiles were measured by a GC-2025 Plus gas chromatograph (Shimadzu, Japan) according to Woortman et al. [70].

### Gravimetric analysis of biomass and lipids

Dry cell weight (DCW) was determined by pelleting 2 mL samples (10,000 g, 10 min), washing cells once with 2 mL ddH_2_O, and lyophilized in pre-weighed microtubes. Intracellular total lipid weight was obtained by extraction using chloroform and methanol according to the protocol of Bligh–Dyer [71]. The harvested cells were washed with ddH_2_O and destructed by a high-pressure homogenizer (Mulsiflex C3, Avestine, Canada), followed by two times sequential solvent extraction using Folch solution incubated for 2 h and 1 h, respectively. The chloroform layer containing yeast lipids was aspirated under a nitrogen stream and the lipids were weighed. The percentage of lipid content and total lipid was calculated based on Eq. 1 and Eq. 2

1 $$\begin{aligned} & Lipid\,~content~\,\% ~w/w \\ &\quad= \frac{{w~\,obtained\,~lipid\,\left( g \right)}}{{w~\,obtained\,~dried~\,biomass\,~\left( g \right)}} \times 100 \end{aligned}$$

2 $$\begin{aligned} & Total\,~lipid\,\left( {lipid\,~concentration~\,g/L} \right) \\ &\quad= \frac{{w\,~lipid\,~obtained~\,\left( g \right)}}{{Volume\,~culture\,\left( L \right)}} \end{aligned}$$

The Biomass yield carbon: carbon efficiency) was calculated based on Eq. 3

3 $$\begin{aligned} & Biomass\,~yield\,~\left( {Growth\,~efficiency} \right)~\,g/g \\ &\quad = \frac{{w~\,biomass~\,synthesized\,~\left( g \right)}}{{w~\,sugar~\,consumed\,~\left( g \right)}} \end{aligned}$$

The lipid yield (substrate to lipid conversion rate), and lipid productivity were calculated based on Eqs. 4, and 5 respectively.

4 $$Lipid~\,yield~\,g/g = \frac{{w~\,lipid~\,obtained~\,\left( g \right)}}{{w\,~substrate\,~consumed~\,\left( g \right)}}$$

5 $$\begin{aligned} & Lipid\,~productivity\,~g/Lh \\ &\quad = \frac{{w~\,lipid~\,obtained~\,~\left( g \right)}}{{V\,~culture\left( L \right) \times ~incubation~\,time~\,\left( h \right)}} \end{aligned}$$

### Sugar analysis

Sugar analysis was carried out using an Agilent 1260 Infinity II LC system with quintenary pump and equipped with Diode Array (DA) and Refractive Index (RI) detectors. In this method, a Rezex ROA-organic H + 8% column from Phenomenex was used (300 × 7.8 mm). The isocratic mobile phase (5 mM H2SO4) was pumped at a flow rate of 0.5 mL/min. The run time was 60 min. The oven temperature was set at 70 °C, and the measurement was done by RID at 40 °C without cooling. The injection volume was 10 µl. All the sugars used for calibrations were obtained from Sigma, Germany.

The percentage of substrate consumed was calculated according to Eq. 6.

6 $$\begin{aligned} & Substrate~\,consumed{\kern 1pt} ~\% ~w/w~\,at\,~time~\,point ~\,~x \\ &\quad= \frac{{w\,~substrate~\,consumed\,~at\,~time~\,point ~\,x{\kern 1pt} ~\left( g \right)}}{{w~\,total~\,available\,~substrate~\,at~\,the\,~start~\,point {\kern 1pt} ~\left( g \right)}} \times 100\end{aligned}$$

### Element analysis

Elemental analysis (C, H, N, S) was done using a Euro EA CHNS elemental analyzer (HEKAtech Ltd.), based on dynamic spontaneous combustion in the Sn boat at approximately 1800 °C with subsequent gas chromatographic separation and detection using a thermal conductivity detector (TCD).

## Supplementary Information

**Additional file 1:** **Figure S1.** Growth curves of all strains in each medium.

**Additional file 2: Table S1.** Specific sugar consumption in complex media. **Table S2**. The fatty acid profile of each strain in different media. **Table S3** Measurement of dry cell weights for each experiment throughout the cultivation. **Table S4** Lipid contents determined on a dry weight basis for each yeast strain. **Table S5** The compositional analysis of corn steep liquor powder. **Table S6** Overview of lipid contents and lipid concentrations measured in previous studies compared with the current study.

## Data Availability

The datasets supporting the conclusions of this article are included within the article [and its Additional information files].

## Abbreviations

SCO: Single cell oil
WSH: Wheat straw hydrolysate
BAH: Brown algae hydrolysate
CSL: Corn steep liquor
MNM: Minimal nitrogen medium
MNM-Glu: Minimal nitrogen medium containing glucose
MNM-Xyl: Minimal nitrogen medium containing xylose
MNM-Man: Minimal nitrogen medium containing mannitol
MPM-GlcNAc: Minimal phosphate medium containing N-acetylglucosamine
GC-FID: Gas chromatography-flame ionization detection
DCW: Dry cell weight
YO: Yeast oil
h: Hours
FA: Fatty acid
w: Weight
V: Volume
TAGs: Triglycerides
