# Supplementary figures and images for: Oleaginous yeasts- substrate preference and lipid productivity: a view on the performance of microbial lipid producers
Source: Microb Cell Fact. 2021 Dec 7;20:220. doi: 10.1186/s12934-021-01710-3 (PMC8650408; doi:10.1186/s12934-021-01710-3)

—■— C. oleaginosus    —●— R. glutinis    —▲— T. asahii    —▼— R. mucilaginosa    —◆— R. toruloides

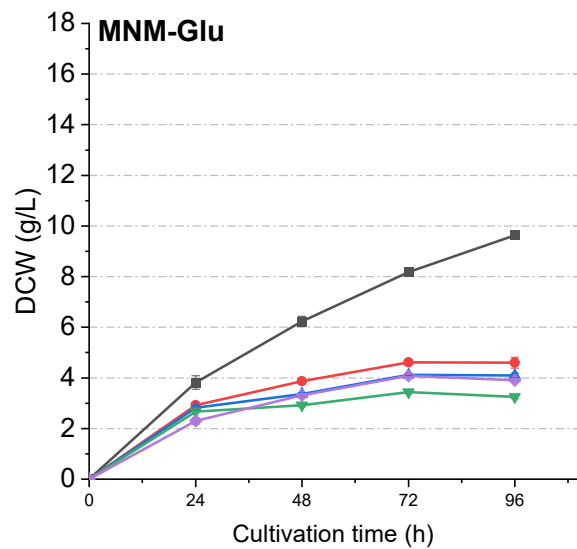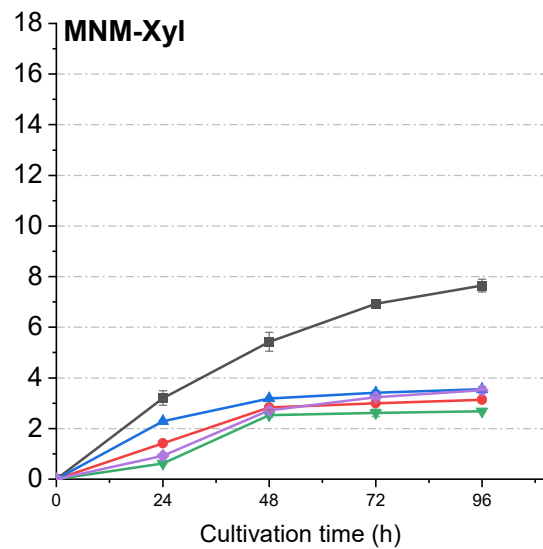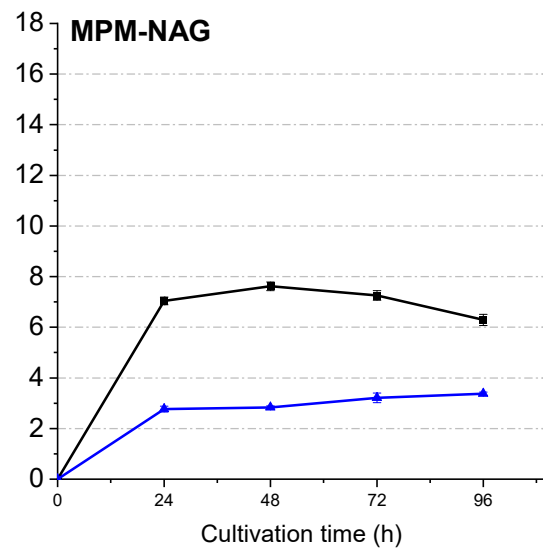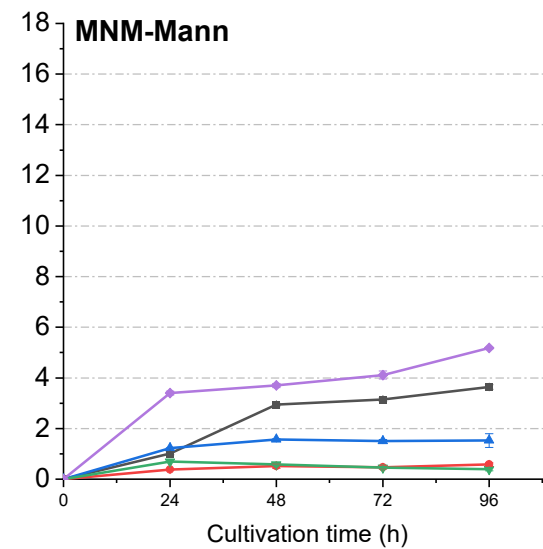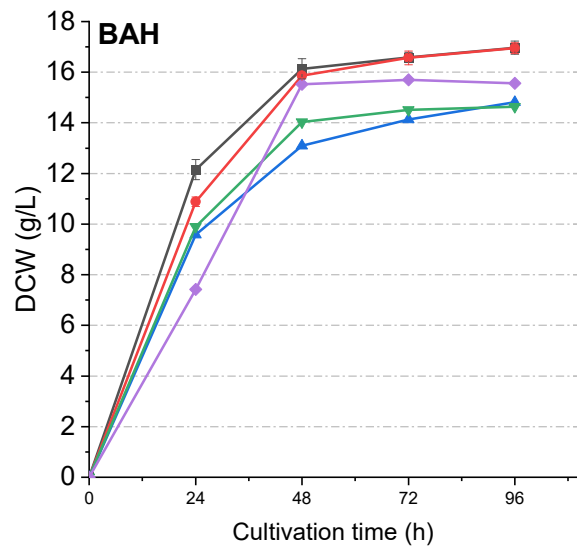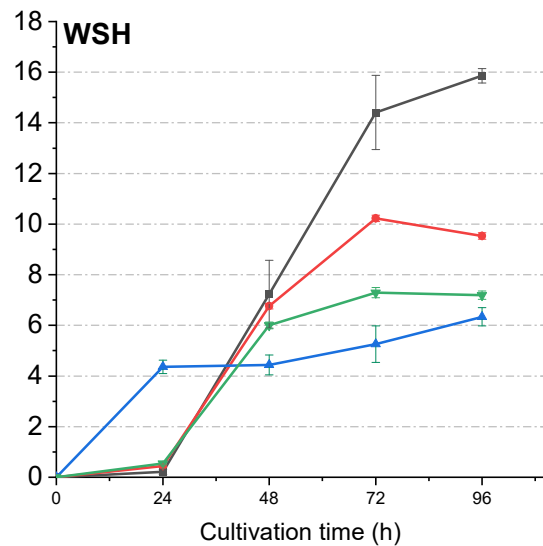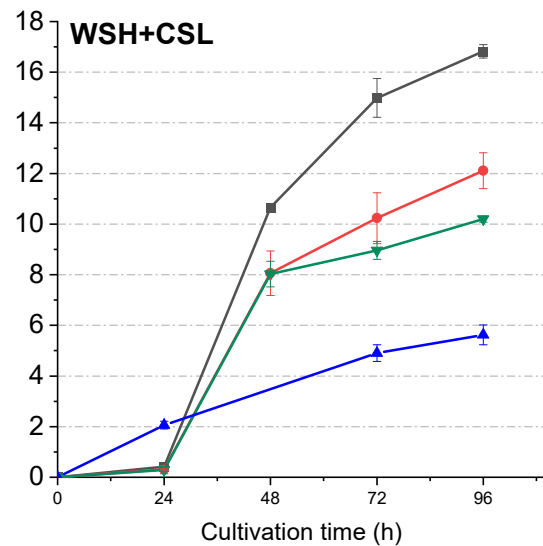

Supplement: Supplementary file 1 — Additional file 1: Figure S1. Growth curves of all strains in each medium. [file 12934_2021_1710_MOESM1_ESM.pdf]
